# **α**2,6 Sialylation mediated by ST6GAL1 promotes glioblastoma growth

**DOI:** 10.1172/jci.insight.158799

**Published:** 2022-11-08

**Authors:** Sajina GC, Kaysaw Tuy, Lucas Rickenbacker, Robert Jones, Asmi Chakraborty, C. Ryan Miller, Elizabeth A. Beierle, Vidya Sagar Hanumanthu, Anh N. Tran, James A. Mobley, Susan L. Bellis, Anita B. Hjelmeland

**Affiliations:** 1 Department of Cell, Developmental and Integrative Biology,; 2 Department of Pathology,; 3 Department of Surgery, and; 4 Department of Medicine, University of Alabama at Birmingham, Birmingham, Alabama, USA.; 5 DataGrata LLC, Chicago, Illinois, USA.; 6 Department of Anesthesiology, University of Alabama at Birmingham, Birmingham, Alabama, USA.

**Keywords:** Cell Biology, Oncology, Brain cancer, Glycobiology

## Abstract

One of the least-investigated areas of brain pathology research is glycosylation, which is a critical regulator of cell surface protein structure and function. β-Galactoside α2,6-sialyltransferase (*ST6GAL1*) is the primary enzyme that α2,6 sialylates N-glycosylated proteins destined for the plasma membrane or secretion, thereby modulating cell signaling and behavior. We demonstrate a potentially novel, protumorigenic role for α2,6 sialylation and *ST6GAL1* in the deadly brain tumor glioblastoma (GBM). GBM cells with high α2,6 sialylation exhibited increased in vitro growth and self-renewal capacity and decreased mouse survival when orthotopically injected. α2,6 Sialylation was regulated by *ST6GAL1* in GBM, and *ST6GAL1* was elevated in brain tumor-initiating cells (BTICs). Knockdown of *ST6GAL1* in BTICs decreased in vitro growth, self-renewal capacity, and tumorigenic potential. *ST6GAL1* regulates levels of the known BTIC regulators PDGF Receptor β (*PDGFRB*), Activated Leukocyte Cell Adhesion Molecule, and Neuropilin, which were confirmed to bind to a lectin-recognizing α2,6 sialic acid. Loss of *ST6GAL1* was confirmed to decrease *PDGFRB* α2,6 sialylation, total protein levels, and the induction of phosphorylation by PDGF-BB. Thus, *ST6GAL1*-mediated α2,6 sialylation of a select subset of cell surface receptors, including *PDGFRB*, increases GBM growth.

## Graphical abstract

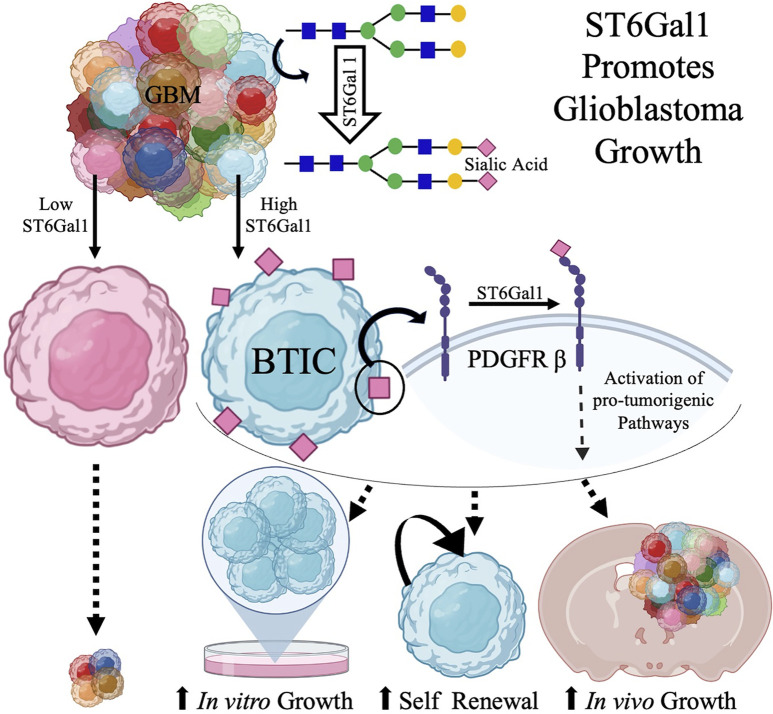

## Introduction

Glioblastoma (GBM) is one of the most aggressive and fatal cancers with a median survival of less than 15 months past diagnosis (1, 2). Contributing to treatment failures and disease progression is the highly heterogeneous nature of GBMs, including a subset of GBM cells called brain tumor-initiating cells (BTICs). BTICs have similarities to neural progenitors including expression of the stem cell marker *SOX2* and the ability to self-renew in neurosphere formation assays, but BTICs can form tumors when orthotopically injected (3–10). BTICs are thought to be a major cause of disease recurrence, and it is therefore imperative that the cellular mechanisms involved in BTIC maintenance are elucidated. While there are many studies dedicated to understanding the genome and proteome of GBMs and BTICs, studies of glycosylation as a post-translational modification are limited, even though altered cell surface glycosylation was one of the earliest modifications observed in malignant neoplastic progression.

Among the various glycosyltransferases present in human cells, *Golgi* sialyltransferase β-galactoside α2,6-sialyltransferase 1 (*ST6GAL1*) and 2 (*ST6GAL2*) add sialic acid residues in α2,6 linkage to membrane-bound and secreted N-glycosylated proteins (11). Due to the position and negative charge of sialic acid, α2,6 sialylation can alter conformation, clustering, and retention of glycoproteins (12–14). Altered glycosylation is a cancer hallmark, and *ST6GAL1* is one of the main glycosyltransferases upregulated in malignancies. In epithelial cancers, *ST6GAL1* has been shown to regulate α2,6 sialylation and impart tumor-initiating cell (TIC) phenotypes, including sustained proliferative capacity, upregulation of TIC markers (*CD133, ALDH1*), sphere formation capacity, resistance to cell death induced by chemotherapies, growth factor withdrawal, and inflammatory mediators (15–24). To date, *ST6GAL1* is known to exert its biological effects in cancer cells by modulating the function of select receptors including TNF Receptor 1 (*TNFR1*) and *EGFR*, leading to the activation of transcription factors such as NF-κB (25, 26). While these pathways are known to play critical roles in brain tumors, the levels, or ability, of *ST6GAL1*, *ST6GAL2*, or α2,6 sialylation to modulate BTIC signaling or maintenance to increase glioma growth has not been investigated.

In contrast to the protumorigenic role of *ST6GAL1* in pancreatic and ovarian cancers (among others), prior reports suggested that *ST6GAL1* is suppressed and plays a tumor-suppressive role in GBM (27, 28). The studies used standard human glioma cell lines, primarily U373MG, propagated in media containing FBS, which is known to promote cell differentiation. Studies herein show that *ST6GAL1* levels are much higher in GBM BTICs than in differentiated GBM cells. Importantly, we define a potentially novel protumorigenic role for *ST6GAL1* in GBM due, in part, to the regulation of α2,6 sialylation in BTICs. We have assessed α2,6 sialylation, specifically by *ST6GAL1*, using patient-derived xenografts (PDXs) representing different GBM subtypes. Using a lectin that binds α2,6 sialic acids, we demonstrated that α2,6 sialylation^hi^ GBM cells were enriched for growth in vitro and in vivo. We determined that *ST6GAL1* levels were high in BTICs and, using lentivirus expressing nontargeting or *ST6GAL1*-directed shRNAs, we demonstrated a critical role for *ST6GAL1* in α2,6 sialylation in GBMs. In this study, we have also identified specific N-glycosylated proteins that are sialylated and whose expression is regulated by *ST6GAL1*. Our findings define α2,6 sialylation and *ST6GAL1* as central regulators of GBM growth and BTIC maintenance. These results are important because *ST6GAL1* inhibitors are in development, although specific sialyltransferase inhibitors are not yet available. Furthermore, defining α2,6 sialylated proteins in GBMs or BTICs may identify new biomarkers for the disease or cellular subsets.

## Results

### GBM cells with elevated α2,6 sialylation have increased growth in vitro and in vivo.

**Figure 1:**
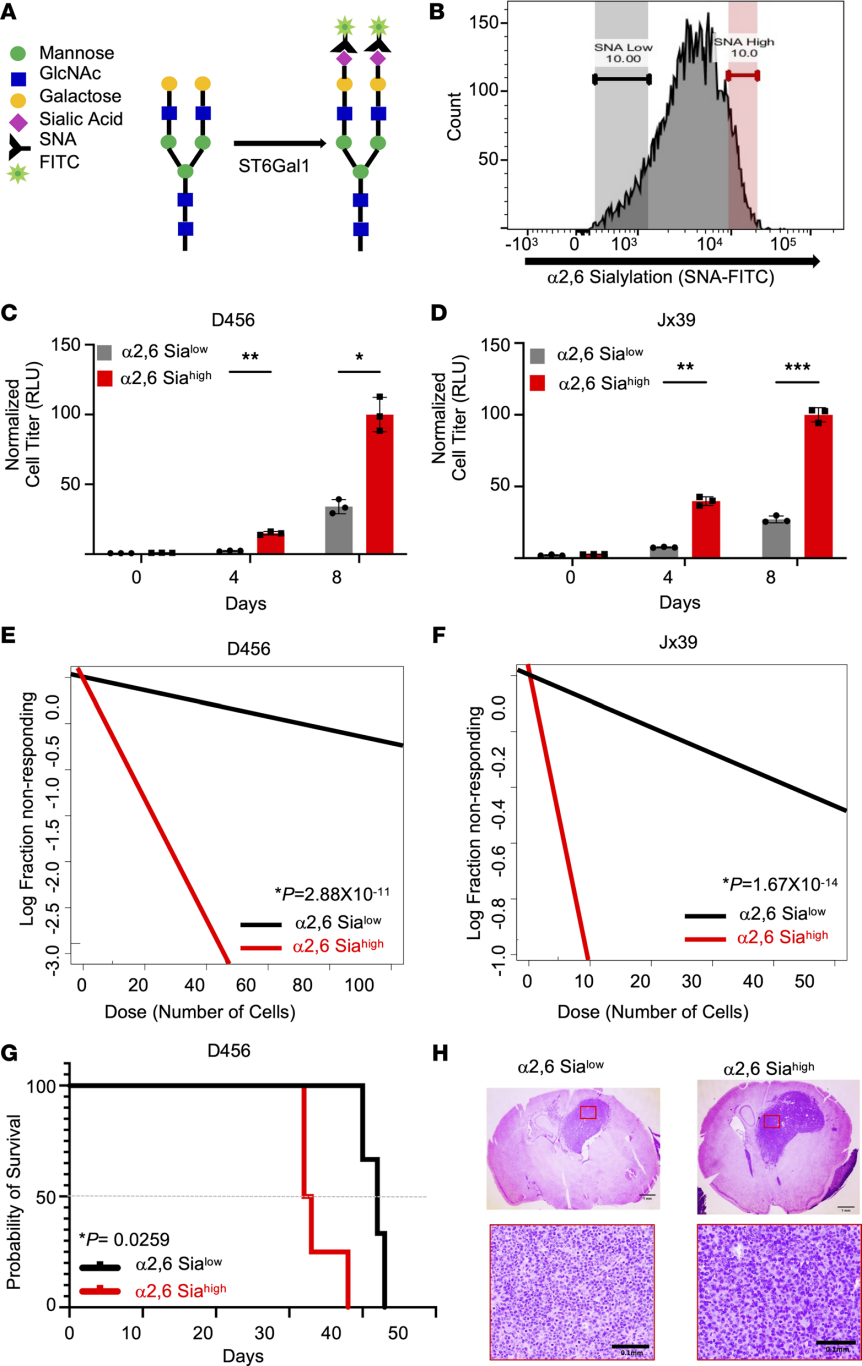
α2,6 Sialylation increases GBM growth and self-renewal. (**A**) Schematic of SNA, lectin with high affinity for α2,6 sialic acid, tagged with FITC as used for flow cytometry. (**B**) Representative histogram using SNA-FITC for FACS to sort SNA^hi^ or α2,6 sialylation^hi^ (highest 10% intensity) and SNA^lo^ or α2,6 sialylation^lo^ (lowest 10% intensity) cells. A total of 1,000 α2,6 sialylation^hi^ versus α2,6 sialylation^lo^ cells isolated from (**C**) D456 and (**D**) JX39 GBM PDXs were directly plated during FACS, and growth was measured over time using CellTiter-Glo 2.0 (luminescence, RLU). Individual data points are shown with the error bars as mean ± SD (n = 3). *P < 0.05; **P < 0.01; ***P < 0.001, 2-way ANOVA with Tukey’s multiple comparisons test. The experiments were repeated in 3 independent biological replicates. Data from 1 representative experiment are shown. Differences in self-renewal and BTIC frequencies were determined using in vitro limiting dilution assays with α2,6 sialylation^hi^ versus α2,6 sialylation^lo^ cells isolated from (**E**) D456 and (**F**) JX39 GBM PDXs. Each group was plated in decreasing number of cells (100, 50, 10, 5, and 1 cell per well). Extreme limiting dilution analysis (ELDA) was done using the software (http://bioinf.wehi.edu.au/software/elda/). *P* values were calculated from χ^2^ analysis of group comparisons. The experiments were repeated in 3 independent biological replicates. Data from 1 representative experiment are shown. (**G**) Kaplan-Meier survival curves for BALB/c *nu/nu* mice injected orthotopically with 2,500 α2,6 sialylation^hi^ or α2,6 sialylation^lo^ cells isolated from D456 PDX cells and euthanized upon development of neurological signs. *P* value was calculated using log-rank (Mantel-Cox) test. (**H**) Representative histological images of tumors stained with H&E support the presence of brain tumors in mice with neurological signs. Top panels: Image objective = 1.25×; scale bar: 1.0 mm. Bottom panels: Image objective = 20×; scale bar: 0.1 mm.

While TIC maintenance is known to require cell surface glycosylation, the interplay between cell surface protein modification/cell signaling and TIC maintenance, particularly with regard to sialylation, remains largely understudied (16, 29–31). To first determine if there were any functional consequences for α2,6 sialylation in GBM, we utilized Sambucus nigra agglutinin (SNA), a lectin that specifically binds to terminal Gal- or GalNAc-linked α2,6-linked sialic acid (Figure 1A). Using SNA conjugated to FITC with FACS, GBM cells were isolated directly from PDXs and sorted for α2,6 sialylation (Figure 1B). GBM cells in the highest tenth percentile of intensity for SNA binding were identified as α2,6 sialylation^hi^ and those in the lowest tenth percentile of intensity for SNA binding as α2,6 sialylation^lo^. In 2 different xenografts, α2,6 sialylation^hi^ GBM cells had significantly higher growth rates than α2,6 sialylation^lo^ cells as determined via cell titer assays (Figure 1, C and D) and crystal violet staining (Supplemental Figure 1, A and B; supplemental material available online with this article; https://doi.org/10.1172/jci.insight.158799DS1). As α2,6 sialylation has been linked to pluripotency, we next sought to determine if α2,6 sialylation could impact self-renewal and the percentages of BTICs in GBMs using neurosphere formation assays. Using cells directly isolated from 2 different PDXs and sorted for α2,6 sialylation using SNA-FITC, we determined an enrichment for neurosphere-formation capacity in α2,6 sialylation^hi^ GBM cells (Figure 1, E and F). We next evaluated the importance of α2,6 sialylation for GBM growth in vivo. α2,6 sialylation^hi^ or α2,6 sialylation^lo^ GBM cells were intracranially injected into BALB/c *nu/nu* mice and monitored daily for the development of neurologic signs (Figure 1, G and H). Consistent with the in vitro data, the Kaplan-Meier survival curves revealed that the mice injected with α2,6 sialylation^hi^ cells had significantly decreased survival, demonstrating that α2,6 sialylation promotes GBM growth in vivo. The presence of brain tumors in mice with neurologic signs was confirmed via H&E staining of fixed tissue sections (Figure 1H). Blinded review of these sections or those stained with *Ki67* by a neuropathologist did not indicate substantial pathologic differences at this endpoint. These data defined a novel role for α2,6 sialylation in GBM growth and self-renewal.

### α2,6 Sialylation in GBM is regulated by ST6GAL1, which is increased in BTICs.

**Figure 2:**
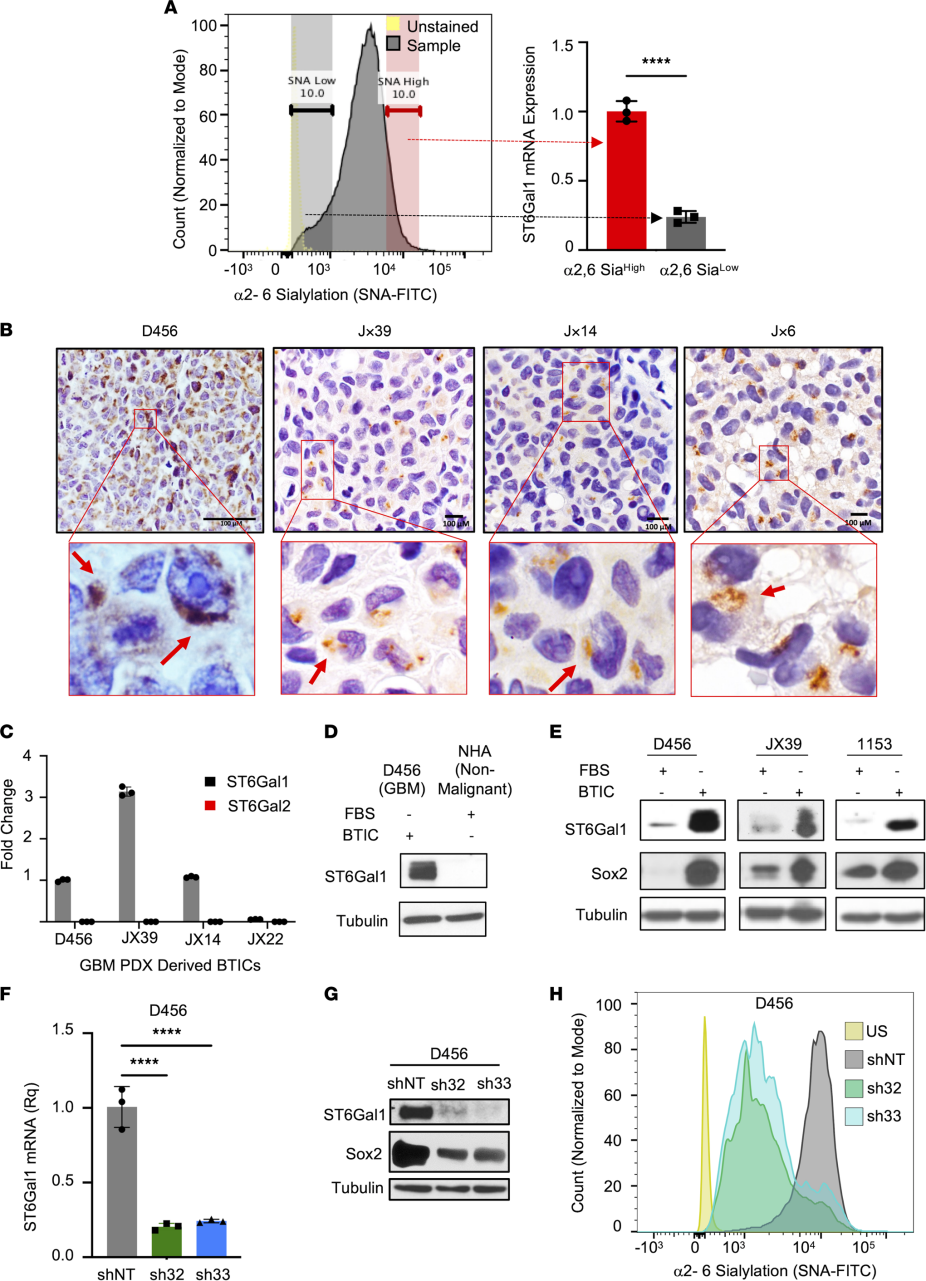
*ST6GAL1* is expressed in GBM and elevated in BTICs to increase α2,6 sialylation. (**A**) Example histogram of FACS with SNA-FITC to identify α2,6 sialylation^hi^ and α2,6 sialylation^lo^ cells that were lysed after sorting and *ST6GAL1* mRNA levels determined using qRT-PCR. Relative quantification (Rq) is normalized to SNA^hi^. Individual data points are shown with the error bars as mean ± SD (*n* = 3). ****P < 0.0001 with 2-tailed *t* test. The experiments were repeated in at least 3 independent biological replicates. Data from 1 representative experiment are shown. (**B**) IHC of *ST6GAL1* in sections of 4 different s.c. human GBM xenografts. From left, D456, JX39, Jx14, and JX6. Image objective D456 40x and JX39, Jx14, and JX6 60x oil immersion. Scale bars: 100 μm. The red box represents the section from which the magnified images were collected. The red arrows indicate punctate *Golgi* staining for *ST6GAL1*. (**C**) mRNA levels of *ST6GAL1* or *ST6GAL2* in BTICs isolated from the indicated GBM xenografts; Rq for individual PDX is normalized to D456. Individual data points are shown with the error bars as mean ± SD (*n* = 3). The experiments were repeated in at least 3 independent biological replicates. Data from 1 representative experiment are shown. (**D**) *ST6GAL1* protein levels in nonmalignant brain cells (NHA) or GBM (D456) were determined via IB. NHA, normal human astrocytes. (**E**) *ST6GAL1* protein levels in BTICs or BTICs differentiated in FBS for 96 hours were determined via IB. Differences in expression of the BTIC marker *SOX2* were used as control. The experiments were repeated in at least 3 independent biological replicates. Data from 1 representative experiment are shown. (**F**–**H**) Lentivirus was used to transduce D456 cells with nontargeting control shRNA (shNT) or 2 different shRNA constructs targeting *ST6GAL1* (sh32 and sh33). Cells for analysis and experiments were collected after 24 hours of lentivirus exposure and 72 hours of antibiotic selection. (**F**) KD of *ST6GAL1* mRNA was validated using qRT-PCR. Individual data points are shown with the error bars as mean ± SD (*n* = 3). ****P < 0.0001 with 1-way ANOVA. (**G**) KD of *ST6GAL1* protein was validated with IB. *SOX2* expression in the shNT compared with the KD groups were determined via IB. (**H**) Representative histogram of FACS analysis with SNA-FITC of BTICs with and without *ST6GAL1* modulation in D456 PDX cells demonstrating reduced α2,6 sialylation. The experiments were repeated in at least 3 independent biological replicates. Data from 1 representative experiment are shown.

After verifying that α2,6 sialylation plays an important role in GBM, we next sought to determine the sialyltransferase mediating this effect. The primary enzyme that α2,6 sialylates N-glycosylated proteins in the secretory pathway is *ST6GAL1* (11, 32). *ST6GAL1* is thought to be broadly expressed with its paralog *ST6GAL2*, relatively restricted and at substantially lower levels, but RNA-Seq suggests that both *ST6GAL1* and *ST6GAL2* are expressed in brain tissue (32, 33). While *ST6GAL1* is known to be important for N-glycan sialylation in the mouse brain (34), *ST6GAL1/2* expression and function in the human brain or brain tumors have not been well characterized. Our analysis of data from the Human Protein Atlas RNA-Seq normal tissues project (PRJEB4337) demonstrated higher expression of *ST6GAL1* mRNA compared with *ST6GAL2* in normal brain tissue (Supplemental Figure 2A) (35). Both *ST6GAL1* and *ST6GAL2* were detected in GBMs (regardless of Isocitrate dehydrogenase status) in data from The Cancer Genome Atlas accessed via Gliovis at http://gliovis.bioinfo.cnio.es (36, 37) and/or GBMseq (38) accessed online (Supplemental Figure 2, B–G). While there was no difference in *ST6GAL1* mRNA in GBM compared to nontumor tissue, ST6Gal2 was significantly decreased (Supplemental Figure 2, B and C). We further determined that higher levels of *ST6GAL1* or lower levels of ST6Gal2 correlated with worse glioma patient survival, but there was no difference in survival with a similar mRNA cutoff in only GBM patients (Supplemental Figure 2, H and I; and data not shown). These data suggested *ST6GAL1* as a potential mediator of α2,6 sialylation in GBM, although roles for *ST6GAL2* could not be eliminated. We recognize the limitations of interpreting mRNA data from bulk tumor. However, we did verify that α2,6 sialylation^hi^ cells isolated from GBM PDXs had higher levels of *ST6GAL1* mRNA (Figure 2A). IHC using an extensively validated Ab confirmed that the typical punctate *Golgi* expression of *ST6GAL1* was observed in sections of GBM PDXs, indicating *ST6GAL1* is expressed in vivo (Figure 2B). As *ST6GAL1* is highly expressed in induced pluripotent stem cells (iPSCs), has been implicated in the maintenance of epithelial cancer TICs, and is known to be regulated by the BTIC marker *SOX2* (16, 39–42), we further evaluated *ST6GAL1* and *ST6GAL2* expression in BTICs. Quantitative real-time PCR (qRT-PCR) analysis revealed that, while heterogeneous for the extent of expression, *ST6GAL1* was present in all PDX-derived BTICs tested (Figure 2C). In contrast, *ST6GAL2* mRNA was not detected in the same BTICs (Figure 2C) but was confirmed to be expressed in nontumorigenic but immortalized human astrocytes (Supplemental Figure 2J). *ST6GAL1* protein was higher in BTICs compared with human astrocytes (Figure 2D and Supplemental Figure 3A), and the notion that elevated *ST6GAL1* expression is present in BTICs was further confirmed. When IB was used to compare BTICs and their differentiated counterparts (as determined by differential *SOX2* expression), *ST6GAL1* protein was consistently higher in BTICs (Figure 2E and Supplemental Figure 3, B and C). These data suggested a substantial role for *ST6GAL1* in GBM that could depend on the differentiation state. To evaluate whether α2,6 sialylation is indeed regulated by *ST6GAL1* in GBM, we utilized a lentiviral system to express 2 different shRNAs targeting *ST6GAL1* (sh32 and sh33) or a nontargeting control (shNT) in BTICs isolated from PDXs. We confirmed knockdown (KD) of *ST6GAL1* mRNA and protein using qRT-PCR (Figure 2F) and IB (Figure 2G and Supplemental Figure 3D). These IBs also revealed that targeting *ST6GAL1* reduced expression of *SOX2* (Figure 2G and Supplemental Figure 3E), providing the first suggestion that *ST6GAL1* may be important for BTIC maintenance. Importantly, KD of *ST6GAL1* reduced α2,6 sialylation as determined by FACS analysis with SNA-FITC (Figure 2H). Together, these data indicate that α2,6 sialylation in BTICs is largely imparted by *ST6GAL1*.

### ST6GAL1 is critical for BTIC maintenance.

**Figure 3:**
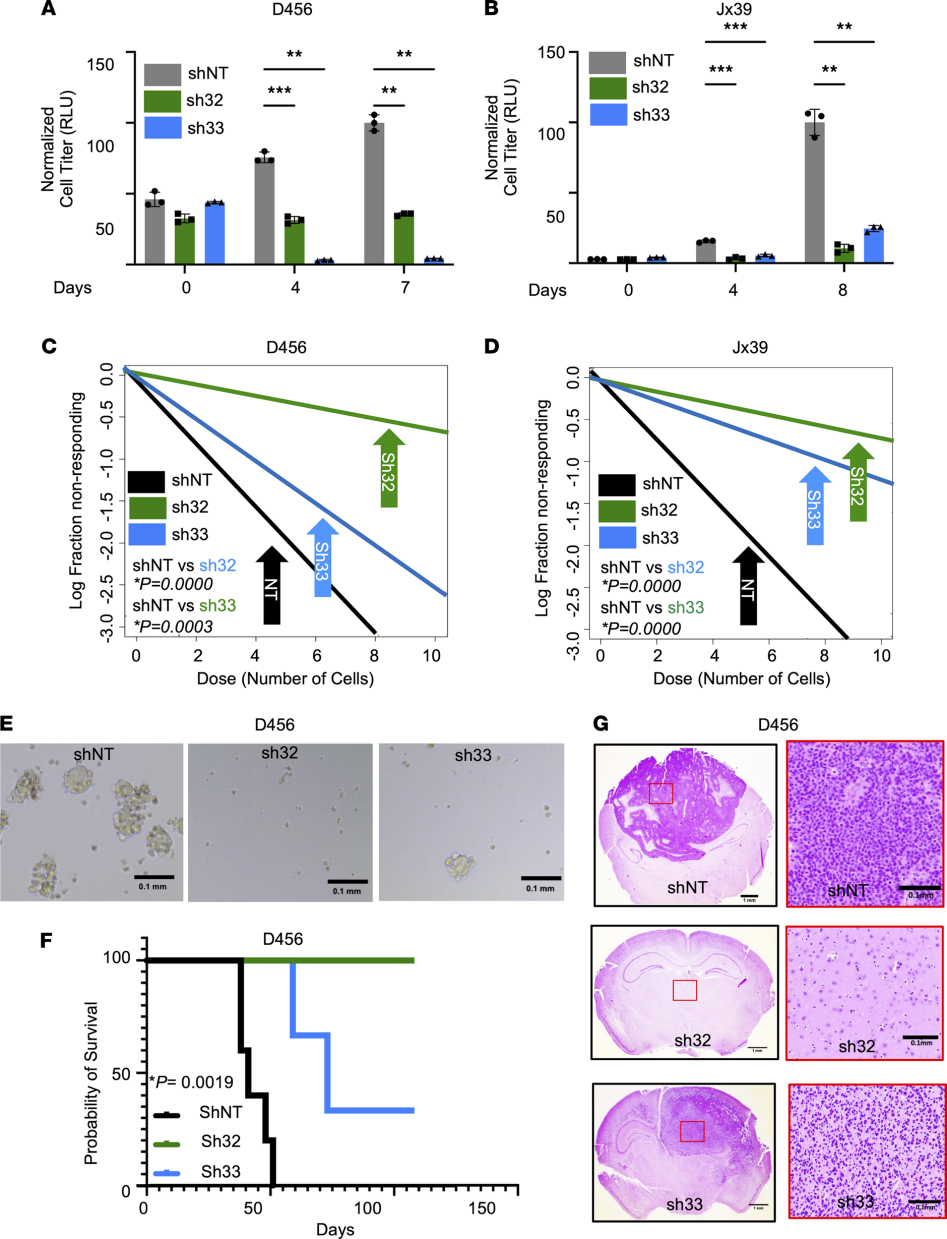
Targeting *ST6GAL1* decreases GBM growth and self-renewal. Growth of (**A**) D456 and (**B**) Jx39 BTICs with (sh32, sh33) and without (nontargeting control, shNT) *ST6GAL1* was measured over time using CellTiter-Glo 2.0 (luminescence, RLU). Individual data points are shown with the error bars as mean ± SD (*n* = 3). **P < 0.01; ***P < 0.001, 2-way ANOVA with Tukey’s multiple comparisons test. The experiments were repeated in 3 independent biological replicates. Data from 1 representative experiment are shown. BTIC frequencies were compared using in vitro limiting dilution assays with (**C**) D456 and (**D**) Jx39 BTICs with and without *ST6GAL1* KD. Each group was plated in decreasing number of cells (100, 50, 10, 5, and 1 cell per well). ELDA was done using the software (http://bioinf.wehi.edu.au/software/elda/). *P* values were calculated from chi-square analysis of group comparisons. The experiments were repeated in at least 3 independent biological replicates. Data from 1 representative experiment are shown. (**E**) Representative images of D456 neurospheres at day 7 at 4× magnification. Scale bar: 0.1 mm. (**F**) Kaplan-Meier survival curves for BalbC nu/nu mice injected orthotopically with 5,000 shNT, sh32, or sh33 D456 BTIC cells and sacrificed upon development of neurological signs. The log-rank test was employed to calculate the indicated *P* value. (**G**) Representative histological images of tumors from **F** stained with H&E support the presence of brain tumors in mice with neurological signs. Left panels: Image objective = 1.25×; scale bar: 1.0 mm. Right panels: Image objective = 20×; scale bar: 0.1 mm.

To determine if loss of *ST6GAL1* could result in phenotypes similar to those in α2,6 sialylation^lo^ cells, we utilized the lentiviral system described above (Figure 2, F–H). BTICs expressing either of 2 different *ST6GAL1* shRNAs had significantly decreased in vitro growth compared with nontargeting controls as determined via cell titer assays (Figure 3, A and B) or crystal violet staining (Supplemental Figure 4, A and B). As *ST6GAL1*-specific inhibitors are not yet available, we next employed an analog of sialic acid to determine the impact of sialyltransferase inhibition on BTIC growth. 3Fax-Peracetyl Neu5Ac inhibits sialyltransferase via the generation of a mimetic of cytosine 5′-monophosphate N-acetylneuraminic acid (CMP-Neu5Ac), the substrate *Golgi* sialyltransferases use to form sialic acid (43). 3Fax-Peracetyl Neu5Ac significantly decreased the growth of BTICs derived from D456 (Supplemental Figure 4, C and D) and Jx39 (Supplemental Figure 4, E and F) PDX. BTICs were substantially more sensitive to the growth inhibitory effects of sialyltransferase inhibition than their non-BTIC counterparts (Supplemental Figure 4, D and F). Additional experiments determined impacts of loss of *ST6GAL1* on BTIC self-renewal; similar to the α2,6 sialylation^lo^ cells, *ST6GAL1* KD cells have significantly decreased neurosphere formation capacity as determined in extreme limiting dilution assays (Figure 3, C–E). Furthermore, loss of *ST6GAL1* significantly inhibited the ability of BTICs to initiate tumors in vivo (Figure 3, F and G). These data indicate that ST6GAL1 promotes BTIC maintenance in vitro and GBM growth in vivo and defines a novel protumorigenic role for *ST6GAL1* and α2,6 sialylation in GBM.

### ST6GAL1 regulates a select subset of cell surface proteins known to regulate TIC maintenance.

**Figure 4:**
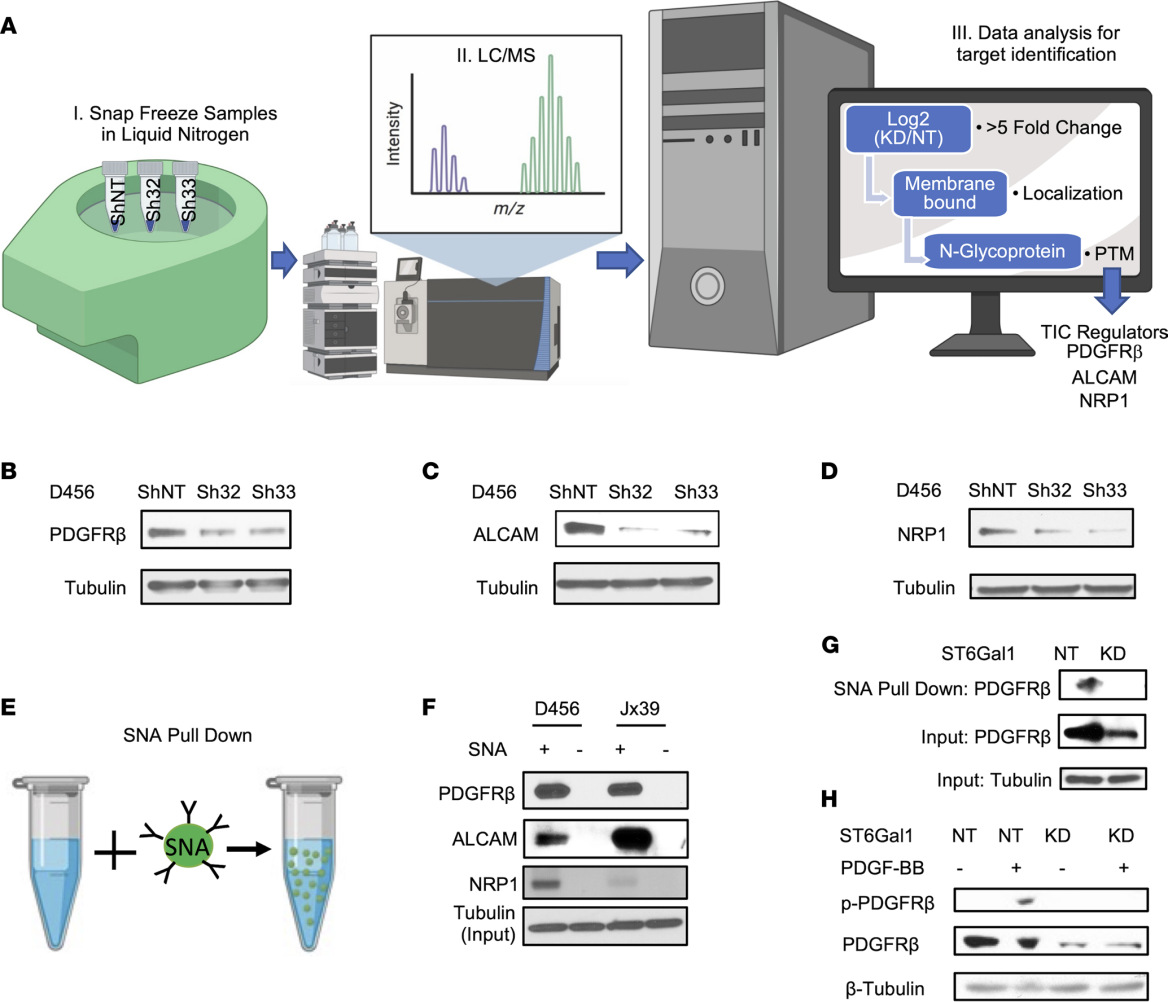
*ST6GAL1* targeting decreases levels of a subset of N-glycoproteins that are known BTIC regulators. (**A**) Schematic of proteomic analysis of D456 BTICs with and without *ST6GAL1* KD (*n* = 4 for each group of shNT, sh32, and sh33). IB with samples independent of the proteomic analysis verified that successful targeting *ST6GAL1* resulted in decreased (**B**) *PDGFRB*, (**C**) *ALCAM*, and (**D**) *NRP1* protein. (**E**) Schematic of pulldown using SNA-bound Agarose beads. (**F**) SNA pulldown and protein A/G bound agarose beads as a control demonstrated that *PDGFRB*, *ALCAM,* and *NRP1* were targets for α2,6 sialylation. (**G**) SNA pulldown of D456 PDX cells with *ST6GAL1* KD compared with NT, illustrating differential pulldown of *PDGFRB*. (**H**) PDGF-BB–induced (10 minutes) activation of *PDGFRB* in D456 GBM PDX cells with *ST6GAL1* KD compared with NT; IB for p*-PDGFRB* and total *PDGFRB*. The experiments were repeated in at least 3 independent biological replicates. Data from 1 representative experiment are shown.

Having determined that *ST6GAL1* had a protumorigenic biological role in GBM, we next sought to define the molecular mechanisms through which *ST6GAL1* could increase GBM growth. Through the addition of the negatively charged sialic acid, *ST6GAL1* is known to modulate many aspects of glycoprotein structure and function, including protein turnover (12–14). While molecular effects of *ST6GAL1* have not been well-studied in BTICs, studies in other tumor types have demonstrated sialylation of a select group of cell surface molecules that are known to play critical roles in brain tumors. Thus, we expected that *ST6GAL1* could regulate the levels of a subset of cell surface proteins and, therefore, performed proteomics of lysates from BTICs with and without *ST6GAL1* KD (Figure 4A). Proteins were identified via mass spectrometry and differentially expressed proteins determined as those with 5-fold or greater positive or negative log_2_ fold changes (Table 1). We focused on proteins that are known to be N-glycosylated. To further prioritize targets for validation, this list was interrogated for known GBM, BTIC, and TIC regulators (Table 1). Through this process, we identified PDGF Receptor β (*PDGFRB*), Activated Leukocyte Cell Adhesion Molecule (*ALCAM*, CD166), and Neuropilin (*NRP1*) as top potential candidates. Regulation of these N-glycoproteins by sialyltransferases has not yet been studied, nor have these proteins been investigated as mediators of *ST6GAL1* effects including those in TICs. While it is likely to be beneficial to further investigate *ST6GAL1* roles in the activity of known targets with key roles in GBM, those targets, including *EGFR*, were not identified as differentially expressed in our analysis as described above. In samples isolated separately from those used for proteomics, we validated that KD of *ST6GAL1* decreased expression of *PDGFRB* (Figure 4B and Supplemental Figure 5A), *ALCAM* (Figure 4C and Supplemental Figure 5B), and *NRP1* (Figure 4D and Supplemental Figure 5C). To determine if this subset of N-glycoproteins was α2,6 sialylated, we performed pulldowns using SNA bound to agarose (Figure 4E). In lysates from BTICs isolated from 2 different PDXs, *PDGFRB, ALCAM*, and *NRP1* associated with SNA agarose beads, but not controls, indicating that these proteins are targets for α2,6 sialylation (Figure 4F and Supplemental Figure 5, D–F). Considering the known importance for *PDGFRB* in GBM growth, we also further explored the impact of *ST6GAL1* on *PDGFRB* and its phosphorylation. Using SNA pulldowns in lysates collected from nontargeting and *ST6GAL1* KD cells, we confirmed that α2,6 sialylation levels of *PDGFRB* were diminished with loss of *ST6GAL1* (Figure 4G and Supplemental Figure 5, G and H). Treatment of nontargeting and *ST6GAL1* KD cells with the *PDGFRB* ligand PDGF-BB also demonstrated that KD of *ST6GAL1* resulted in decreased phosphorylation of *PDGFRB* (Figure 4H and Supplemental Figure 5, I and J). As total levels of *PDGFRB* were decreased as expected based on our proteomics screen, we normalized phospho- to total *PDGFRB* and confirmed a significant decrease (Supplemental Figure 5, I and J). Thus, *ST6GAL1* is a critical regulator of *PDGFRB* signaling whose protumorigenic role in GBM was previously unrecognized. The potentially novel finding that *ST6GAL1* post-translationally modifies critical TIC regulators further implicates *ST6GAL1*-mediated sialylation as an important regulator of BTIC maintenance.

## Discussion

Sialylation is an important post-translational modification that is highly understudied in the brain and in brain tumors, including GBM. We find that α2,6 sialylation and *ST6GAL1* are protumorigenic in GBM. Reports from the Moskal group using GBM cell lines in differentiation-promoting conditions suggested that *ST6GAL1* levels were low in GBM and that loss of *ST6GAL1* would be protumorigenic (27, 28). Our data confirmed that in differentiating conditions the levels of *ST6GAL1* protein were low, but we determined elevated *ST6GAL1* in BTICs. We also observed elevated *ST6GAL1* protein in BTICs compared with nontumor brain cells. We found that enrichment for α2,6 sialylation increased GBM growth in vitro and in vivo in association with increased self-renewal as evaluated through neurosphere formation. α2,6 Sialylation was mediated by *ST6GAL1* and genetic targeting of *ST6GAL1* decreased GBM growth and self-renewal. While the extent of *ST6GAL1* KD appeared similar between the 2 shRNAs used at the mRNA level, results often suggested a greater biological effect of shRNA 32 that correlated with changes in the extent of reduction in sialylation. For example, while both shRNA 32 and shRNA 33 significantly reduced α2,6 sialylation and tumor growth, the effects were most substantial for shRNA 32. Together, our findings demonstrate that *ST6GAL1*-mediated α2,6 sialylation is critical for BTIC maintenance and GBM growth and suggest the translational potential of targeting *ST6GAL1* or α2,6 sialylation. While *ST6GAL1*-specific inhibitors are in development but are not yet available (44–50), sialyltransferase inhibitors have been identified and continue to be developed. We found that inhibiting sialyltransferase activity with 3Fax-Peracetyl Neu5Ac decreased BTIC growth at concentrations that remained ineffective in non-BTICs, which expressed lower levels of *ST6GAL1*. Thus, targeting of *ST6GAL1* may offer benefits for GBM treatment in the future, particularly if it led to increased death of therapy resistant BTICs.

TIC phenotypes and signaling have been informed by results in non-neoplastic stem cells and iPSCs. For *ST6GAL1*, the literature suggests important roles in both the stem cell niche and during reprogramming (42). *ST6GAL1* is present in the base of colon crypts, a well-characterized stem cell niche (29). *ST6GAL1* is elevated during iPSC induction where it is critical for the acquisition of stem cell phenotypes (42). Indeed, somatic cells displayed mostly α2,3 sialylation, whereas iPSCs had high levels of α2,6 sialylation (51). Reprogramming involves the Yamanaka and neural stem cell (and BTIC) transcription factor *SOX2*, which we demonstrated regulates *ST6GAL1* expression (40). Data in this report further demonstrate that *ST6GAL1*, in turn, regulates *SOX2* in BTICs (Figure 2G). Thus, a *SOX2*-*ST6GAL1* feedforward loop that regulates the glycosylation state of GBM cells may exist in BTICs. *ST6GAL1* regulation of *SOX2* may be an indirect outcome of an overall change in the stem cell state. However, *ST6GAL1*-mediated sialylation of cell surface receptors that signal to control *SOX2* transcription could provide a more direct link between the 2 molecules. If true, this could be an important mechanism through which *ST6GAL1* regulates a neural stem cell or neural stem cell-like state in normal and neoplastic cells, respectively.

Although *ST6GAL1* is known to alter the function of a subset of cell surface proteins that have established roles in tumor biology, the molecular mechanisms through which *ST6GAL1* mediates protumorigenic effects remain to be fully elucidated. Our proteomics analysis and SNA pulldowns identified *PDGFRB, ALCAM*, and *NRP1* as potentially novel targets for *ST6GAL1* that are sialylated. *PDGFRB* has important roles in GBM, including in BTICs, where it is elevated and promotes BTIC maintenance, invasion, and tumorigenic potential (52). Similarly, *ALCAM* was suggested as a BTIC marker as *ALCAM* was highly expressed in BTICs where it promoted neurosphere formation capacity and tumor growth while also increasing GBM invasion and metastasis to the brain (53, 54). BTICs also express *NRP1* which increases BTIC marker expression, neurosphere formation capacity, migration, and tumor growth (55). These data support known protumorigenic roles for the *VEGF-NRP1* signaling axis*, ALCAM*, and *PDGFRB* in GBM and other tumors, including in therapy-resistant tumor cell subsets that are likely to be enriched for TICs (56–64). Thus, the molecules we have identified as *ST6GAL1* targets in GBM may mediate *ST6GAL1* effects in other cancers as well.

*PDGFRB, ALCAM*, and *NRP1* have multiple N-glycosylation sites and are membrane-bound proteins that go through modification in the secretory pathway. Considering our SNA pulldown results, these established regulators of TICs are likely targets of *ST6GAL1*, which post-translationally modifies proteins in the secretory pathway by adding the terminal sialic acid in trans *Golgi*. Certainly, the SNA pulldown results in lysates from *ST6GAL1* KD cells indicates *PDGFRB* is a target for *ST6GAL1*-mediated sialylation and that *ST6GAL1* regulates *PDGFRB* levels and phosphorylation. While additional studies outside the scope of the current report will be required to define exact mechanisms through which *ST6GAL1* modulates *PDGFRB, ALCAM*, and *NRP1* as well as other proteins, the literature does support a role for *ST6GAL1* in cell surface protein turnover, including through the regulation of cell surface retention, internalization, and degradation. For example, α2,6 sialylation by *ST6GAL1* increases the turnover of cell surface E-cadherin (65), affects cell surface retention of *PECAM* receptor by internalization and degradation (66), and regulates the internalization of the Fas death receptor (39). Through these mechanisms, *ST6GAL1*-mediated sialylation of integrins, growth factor receptors, and death receptors can regulate cell migration, survival, and differentiation state (11, 20, 25, 29, 39, 67). Therefore, decreases in *PDGFRB, ALCAM*, and *NRP1* levels with *ST6GAL1* KD could be due to specific changes in internalization and degradation of these targets that impact BTIC survival and maintenance. We, however, acknowledge the possibility that the effects of *ST6GAL1* could be more global and other surface receptors that are differentially sialylated could transcriptionally change expression of the proteins that we identified in our screen. Furthermore, proteins that are known targets for *ST6GAL1*-mediated sialylation with roles in GBM (such as *EGFR*) would be important to investigate, even though they were not identified as priority targets in our proteomics analysis. Therefore, it is imperative to further probe sialylation and *ST6GAL1* effects in GBM in future studies.

We demonstrated that inhibition of sialyltransferase activity with a small molecule inhibitor decreases BTIC growth (Supplemental Figure 4) and that *ST6GAL1* sialylates *PDGFRB* (Figure 4). These data indicate that *ST6GAL1*-mediated sialylation regulates BTIC maintenance, especially as sialylation-independent roles of *ST6GAL1* have not been characterized. However, it would be beneficial to determine whether a catalytically inactive mutant of *ST6GAL1* would fail to rescue effects of *ST6GAL1* KD. To achieve this goal, the field would benefit from human *ST6GAL1* mutants that are known to alter α2,6 sialylation and impact *ST6GAL1*-regulated biologies, such as growth, survival, migration, and/or invasion in vitro and in vivo. In rat *ST6GAL1*, Meng et al. demonstrated that aa, including N230, C350, C361, H367, and Y366, are required for sialyltransferase activity as demonstrated in a biochemical assay using CMP-Neu5Ac as a donor and N-acetyllactosamine as an acceptor substrate (68). There is homology in these regions with the human sequence, so determining if similar mutations in human *ST6GAL1* would alter α2,6 sialylation in BTICs or other *ST6GAL1*-expressing human cells would be valuable.

While our study has only explored the role of α2,6 sialylation and *ST6GAL1* in BTICs in vitro and in immunocompromised mouse models, we acknowledge the potential importance of *ST6GAL1* and/or *ST6GAL2* in the brain tumor microenvironment. Indeed, data from GBMseq indicates strong expression of *ST6GAL1* in myeloid cells, *ST6GAL1*, and *ST6GAL2* in oligodendrocyte progenitor cells, and *ST6GAL2* in astrocytes (Supplemental Figure 2, F and G). Thus, we may be underestimating the roles for *ST6GAL1* and/or *ST6GAL2* for GBM growth. Importantly, α2,6 sialylation and *ST6GAL1* have several known functions in immunomodulation that could be relevant for GBMs or other cancers, especially as immunotherapies become increasingly used and tested (69, 70). Glycans with α2,6 sialic acids can bind to siglec2 (CD22) to inhibit B cell receptor signaling. *ST6GAL1* is expressed in B cells where it is important for development and immunoglobulin levels, but sialylation of IgG can occur even when *ST6GAL1* is knocked out from B cells as *ST6GAL1* is secreted from the liver (22, 71, 72). Reducing extracellular *ST6GAL1* via liver KO also results in a proinflammatory state linked to changes in macrophages and T cells (73). *ST6GAL1* has also been linked to macrophage survival (13). Inhibition of *ST6GAL1* and α2,6 sialylation was also associated with a proinflammatory state in arthritis (74). As these data may suggest, when *ST6GAL1* was elevated in hepatocarcinoma cells, an immunosuppressive environment was supported: this was due, in part, to inhibition of T cell proliferation (74). *ST6GAL1* is also expressed in human NK cell lines and primary cells, and activation of NK cells with IL2 results in increased α2,6 sialylation. While this increase in sialylation was not associated with an increase in *ST6GAL1* mRNA levels, *ST6GAL1* mRNA and protein are not always correlated and the protein expression of *ST6GAL1* was not fully determined in this study (23). Thus, there are multiple mechanisms through which *ST6GAL1* could impact the immuno-landscape of cancers including GBMs.

In conclusion, these data indicate the understudied importance of post-translational modifications, including sialylation and other types of glycosylation, in GBM. Considering that BTIC characterization may rely on Abs that recognize glycosylated forms of cell surface proteins (such as AC133 for CD133), it is possible that TIC enrichment is selecting for more global differences in glycosylation than currently appreciated. Taken together, our investigation defines a novel role of *ST6GAL1*-mediated α2,6 sialylation in the promotion of GBM growth.

## Methods

### Culture and maintenance of GBM PDX and BTICs.

The GBM PDXs were obtained from Yancey Gillespie and the Brain Tumor Core Facility of the University of Alabama at Birmingham (UAB), Darrel Bigner at Duke University, and Jann Sarkaria at the Mayo Clinic. CSC293T cells were produced and expanded as previously described (4, 5, 9). For dissociation, papain from Worthington Biochemical was used per the manufacturer’s instructions. For in vitro BTIC propagation, DMEM/F12 basal media (catalog 21041-025, Life Technologies) supplemented with EGF, (catalog 300-110P, GeminiBio), FGF (catalog 300-112P, GeminiBio), sodium pyruvate (catalog 11360070, Gibco), penicillin/streptomycin (catalog 15-140-122, Gibco), and GEM21 (a B27 equivalent; catalog 400-161, GeminiBio) were used. To differentiate the BTICs, 10% FBS (catalog PS-FB2, Peak Serum) was added while growth factors and GEM21 were removed.

### Lentiviral gene modulation.

CSC293T cells were transiently cotransfected with psPAX2, pCMV-VSVG, and shRNA constructs using FuGENE HD Transfection Reagent (catalog PRE2312, Promega) as previously reported. Virus titer was determined using Lenti-X qRT-PCR Titration Kit (catalog 740956.50, Takara). Lentivirus-expressing *ST6GAL1* shRNAs (TRCN0000035432 and TRCN0000035433) and nontargeting control shRNA (pLKO.1-TC cloning vector; catalog SHC002) were purchased from Dharmacon. Both shRNA constructs were designed against the *ST6GAL1* coding sequence.

The shRNA sequences were as follows: ST6Gal-I shRNA32: 5′ CCGGCGTGTGCTACTACTACCAGAACTCGAGTTCTGGTAGTAGTAGCACACGTTTTTG 3′; ST6Gal-I shRNA33: 5′ CCGGGCGCTTCCTCAAAGACAGTTTCTCGAGAAACTGTCTTTGAGGAAGCGCTTTTTG 3′.

### mRNA extraction, cDNA generation, and qRT-PCR.

Total mRNA from cells in BTIC media or 96 hours after treatment in differentiation medium was harvested using Qiagen RNeasy Mini Kit (catalog 74106) and synthesized into cDNA using the M-MLV reverse transcriptase cDNA Synthesis Kit (catalog M170A, Promega). qRT-PCR was performed on the generated cDNA with the Taq Man Fast Advanced Master Mix (catalog A44360, Thermo Fisher Scientific). The relative expression of *ST6GAL1* and *ST6GAL2* was measured using *ST6GAL1* FAM/MGB TaqMan Primer (catalog HS00949382_m1, Life Technologies) and *ST6GAL2* FAM/MGB TaqMan Primer (catalog Hs00383641_m1, Life Technologies). The data were analyzed and normalized against housekeeping gene 18S Subunit (catalog Hs99999901_s1, Life Technologies) expression to determine relative expression of target genes.

### IB.

Cells in BTIC media or 96 hours after culture in differentiation medium were harvested and lysed using RIPA Lysis and Extraction Buffer (catalog 89901, Thermo Fisher Scientific). Protein concentration was determined using the BCA assay (catalog 23227, Thermo Fisher Scientific). Prior to electrophoresis on 4%–20% Tris-Glycine Mini Gels (catalog xp04200Box, Invitrogen), protein lysates were denatured with Pierce Lane Marker Reducing Sample Buffer (catalog 39000, Thermo Fisher Scientific). Protein was then transferred to PVDF membranes (catalog SLHV033RS, Thermo Fisher Scientific) and blocked using 5% nonfat milk in TBST or Pierce Protein Free Blocking Buffer (catalog 37571, Thermo Fisher Scientific). The primary Abs for Western blot were *ST6GAL1* (catalog AF5924, R&D Systems), *SOX2* (catalog 561469, BD Biosciences), Tubulin (catalog ab21058, Abcam), *PDGFRB* (catalog 3169, Cell Signaling), Phospho-*PDGFRB* (Tyr751) (catalog 3161, Cell Signaling), *NRP1* (catalog AF3870, R&D Systems), and *ALCAM* (catalog AF656, R&D Systems). HRP-conjugated secondary Abs for Western blot were Anti-Goat (catalog MP-7405, Vector Labs), Anti-Rabbit (catalog A27036, Invitrogen), Anti-Mouse (catalog, A28177, Invitrogen), and Anti-Sheep (catalog HAF016, R&D Systems). SuperSignal West Dura Chemiluminescent (catalog 34076, Thermo Fisher Scientific) reagent was used for chemiluminescent reaction, which was captured using HXR Film (catalog XC6A2, Hawkins X-Ray Supply) and developed in Medical Film Processor (catalog SRX-101A, 105235078, Konika Minolta Medical and Graphic). The developed respective bands on the film were quantified using ImageJ2 (NIH) (75).

### SNA pulldown.

Sambucus Nigra Agglutinin bound Agarose beads (catalog AL-1303-2, Vector Laboratories) were washed twice with ice-cold PBS (catalog 10010049, Thermo Fisher Scientific). Pierce Protein A/G Plus Agarose beads (catalog 20423, Thermo Fisher Scientific) were used as control. After washing 50 μL of SNA Agarose beads or Protein A/G Plus Agarose beads, they were incubated with 500–1,000 μg cell lysates (collected as described above) in a total of 1 mL volume for 4 hours to overnight in a dark cold room (4°C) on a rotator. Beads were washed twice with ice-cold PBS followed by incubation with Pierce Lane Marker Reducing Sample Buffer (catalog 39000, Thermo Fisher Scientific) for 5 minutes at higher than 90°C for 5 minutes. The pulled lysates were subjected to IB as described above.

### Recombinant human PDGF-BB protein treatment.

Cells with indicated modifications and culture conditions were treated with PDGF-BB (catalog 220-BB-010, R&D Systems) reconstituted in 4 mM HCL per manufacturer’s instruction at a final concentration of 5 μg/mL for 10 minutes. Cells were lysed and collected for IB. The appropriate dilution of 4 mM HCL was used as control.

### IHC.

The GBM PDXs propagated intracranially and s.c. were formalin-fixed and paraffin-embedded. The respective tissue samples were incubated overnight at 4°C ST6GAL1 primary Ab (1–5 μg/mL; catalog AF5924, R&D Systems) and IHC was performed as previously described (29). The images were captured using Nikon Eclipse 80i camera and ISCapture software as well as EVOS XL microscope.

### FACS.

Cells from culture or directly isolated the night prior to sorting from GBM xenografts were used for flow cytometry. Cells were washed with cold DMEM:F12 (Gibco) and counted. Cells were resuspended in 90 μL of DMEM:F12 per 7 × 10^6^ cells and incubated with or without SNA-FITC (catalog F-6802-1, EY Laboratories) and sorted by BD-FACS ARIA. Forward and side scatter and viability dyes were also used. Cells were sorted with the assistance of the Flow Cytometry Core at the UAB. The top and bottom 10% were designated as α2,6 sialylation^hi^ and α2,6 sialylation^lo^ populations and were directly sorted into 96-well plates in BTIC medium for experiments or sorted into flow cytometry tubes, pelleted, and lysed.

### Measurement of cell growth.

First, 1 × 10^3^ cells were seeded in 96-well plates containing 100 μL of BTIC medium. Cells were incubated for the indicated number of days at 37°C and total ATP was determined using CellTiter-Glo 2.0 kit (catalog G9243, Promega) in which ATP-driven luminescence corresponds with cell numbers. The luminescence was read using the Biotek synergy H1 microplate reader. For crystal violet growth assay, cells were seeded as described above on the Geltrex-treated plate (catalog A14133-02, Thermo Fisher Scientific) for adherence for the indicated number of days at 37°C. At endpoint, cells were washed twice with PBS (catalog 10010049, Thermo Fisher Scientific) and fixed in 10% buffered formalin (catalog 305-510, Thermo Fisher Scientific). Cells were then incubated with 0.05% crystal violet (catalog S25274B, Thermo Fisher Scientific) for 30 minutes at room temperature, extensively washed in deionized water to remove excess crystal violet and air-dried overnight. Crystal violet absorbed by cells corresponding to cell number in each group were dissolved in 50 μL of 10% acetic acid (catalog A38S-500, Thermo Fisher Scientific) for 15 minutes and absorbance was read at 590 nm using the Biotek synergy H1 microplate reader.

### Neurosphere formation assay.

For in vitro limiting dilution assays, α2,6 sialylation^hi^ and α2,6 sialylation^lo^ or *ST6GAL1* modulated cells were plated in decreasing numbers of cells per well (100, 10, 5, 2, and 1) in 96-well plates containing BTIC medium. The wells containing neurospheres were marked and counted after 14–21 days of incubation. ELDA was performed using software available at http://bioinf.wehi.edu.au/software/elda/

### In vivo tumor initiation assay.

All animal procedures were performed in accordance with the UAB IACUC approved protocols. Animals were housed in a temperature-controlled vivarium with a 14-hour light/10-hour dark cycle at no more than 7 animals per cage. Viable cells were intracranially injected into female athymic nude mice 4–6 weeks of age. A total of 2,500 cells were used for experiments with FACS-sorted cells, whereas 1,000 cells were used for experiments with lentivirus-infected BTICs. Animals were maintained until development of neurological signs (for example, lethargy, ataxia, seizures, and/or paralysis), when brains were collected. Animals without neurologic signs were sacrificed at the termination of the experiment. Harvested brains were fixed in 4% paraformaldehyde and embedded in paraffin and sectioned on slides with subsequent H&E staining at the UAB Tissue Biorepository. Developed slides were imaged with a Nikon Eclipse 80i camera and ISCapture software.

### In silico data analysis.

*ST6GAL1* and *ST6GAL2* gene expression and patient survival data were downloaded from GlioVis (http://gliovis.bioinfo.cnio.es) and plotted to assess expression and survival. The reads per kilobase of transcript per million data for *ST6GAL1* and *ST6GAL2* in brain from Human Protein Atlas RNA-Seq normal tissues project were downloaded from the National Center for Biotechnology Information (https://www.ncbi.nlm.nih.gov/gene/6480) and (https://www.ncbi.nlm.nih.gov/gene/84620). *ST6GAL1* and *ST6GAL2* gene expression data in various cell types present in GBM patient samples via single-cell RNA-Seq were downloaded from GBMSeq (http://www.gbmseq.org).

### Proteomics.

Lysates were prepared in M-Per in quadruplicates and peptide digests separated and analyzed with a Thermo Orbitrap Velos Pro hybrid mass spectrometer equipped with a nano-electrospray source (Thermo Fisher Scientific) similar to our prior report (76). The XCalibur RAW files were converted and mgf files searched using SEQUEST to generate peptide IDs that were filtered using Scaffold (Protein Sciences). Normalized spectral counts were used to calculate fold changes and proteins with greater than 5-fold changes among the nontargeting control and KD samples further analyzed to determine cell surface N-glycoproteins.

### Statistics.

All statistics were performed with GraphPad Prism Version 7 or 9 (GraphPad Software). Both 1- and 2-way ANOVA and multiple *t* tests were performed with Dunnett’s or Tukey’s test for multiple comparisons, and *P* values indicate a confidence level of 95% and significance of 0.05. Correlation analysis was performed using Pearson’s correlation analysis with a CI of 95%. Kaplan-Meier survival curves were compared with the log-rank statistical analysis to determine significant differences in outcome.

### Study approval.

All animal studies were approved by the UAB Institutional Animal Care and Use Committee.

## Author contributions

SGC, SLB, and ABH conceived the project. SGC, AC, CRM, EAB, VSH, JAM, SLB, and ABH designed the experiments. SGC, KT, LR, RJ, AC, VSH, ANT, JAM, and ABH performed experiments and/or analyzed the data generated. SGC and ABH wrote the manuscript with review and approval by all authors. ABH supervised the work.

## Supplementary Material

Supplemental data

## Figures and Tables

**Table 1:**
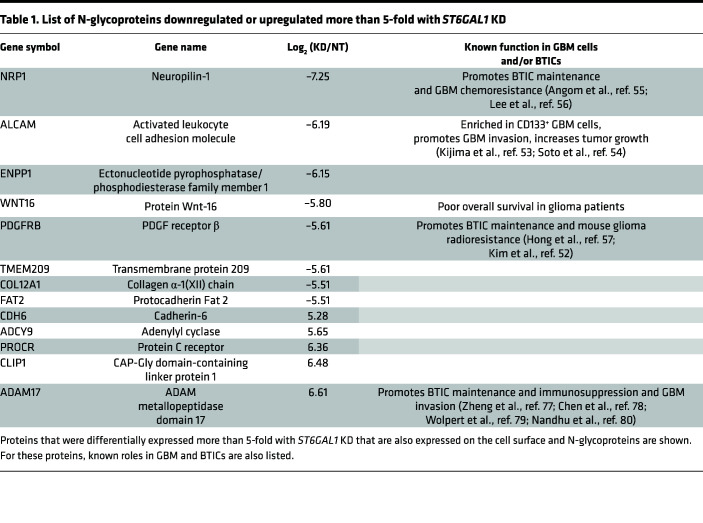
List of N-glycoproteins downregulated or upregulated more than 5-fold with *ST6GAL1* KD

## Version 1 11/08/2022

Electronic publication
