# Supplementary material for: α2,6 Sialylation mediated by ST6GAL1 promotes glioblastoma growth
Source: JCI Insight. 2022 Nov 8;7(21):e158799. doi: 10.1172/jci.insight.158799 (PMC9675560; doi:10.1172/jci.insight.158799)
Supplement: Supplemental data [file jciinsight-7-158799-s272.pdf]

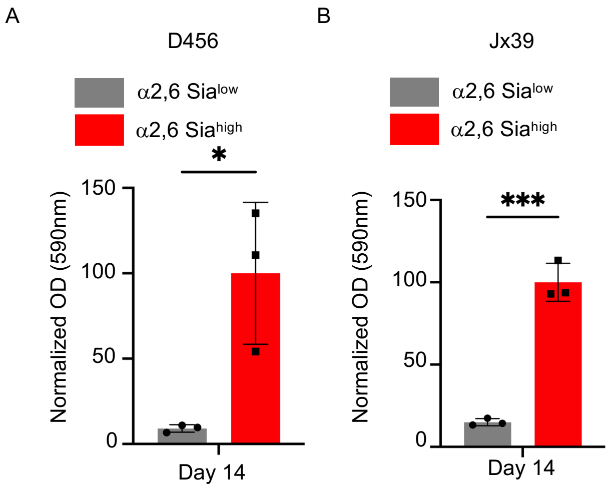

Supplemental Figure 1. α2,6 sialylation increases GBM growth. 1000 α2,6 sialylation^{high} vs α2,6 Sialylation^{low} cells isolated from **(A)** D456 and (**B)** JX39 GBM PDXs were directly plated on 96 well plates coated with geltrex during FACS and growth measured at day 14 using crystal violet staining, absorbance at 590 nm. Individual data points are shown with the errors bars as mean ± SD, (n=3). *P < 0.05; **P < 0.01; ***P < 0.001, 2-way ANOVA with Tukey's multiple comparisons test. The experiments were repeated in three independent biological replicates. Data from one representative experiment are shown.

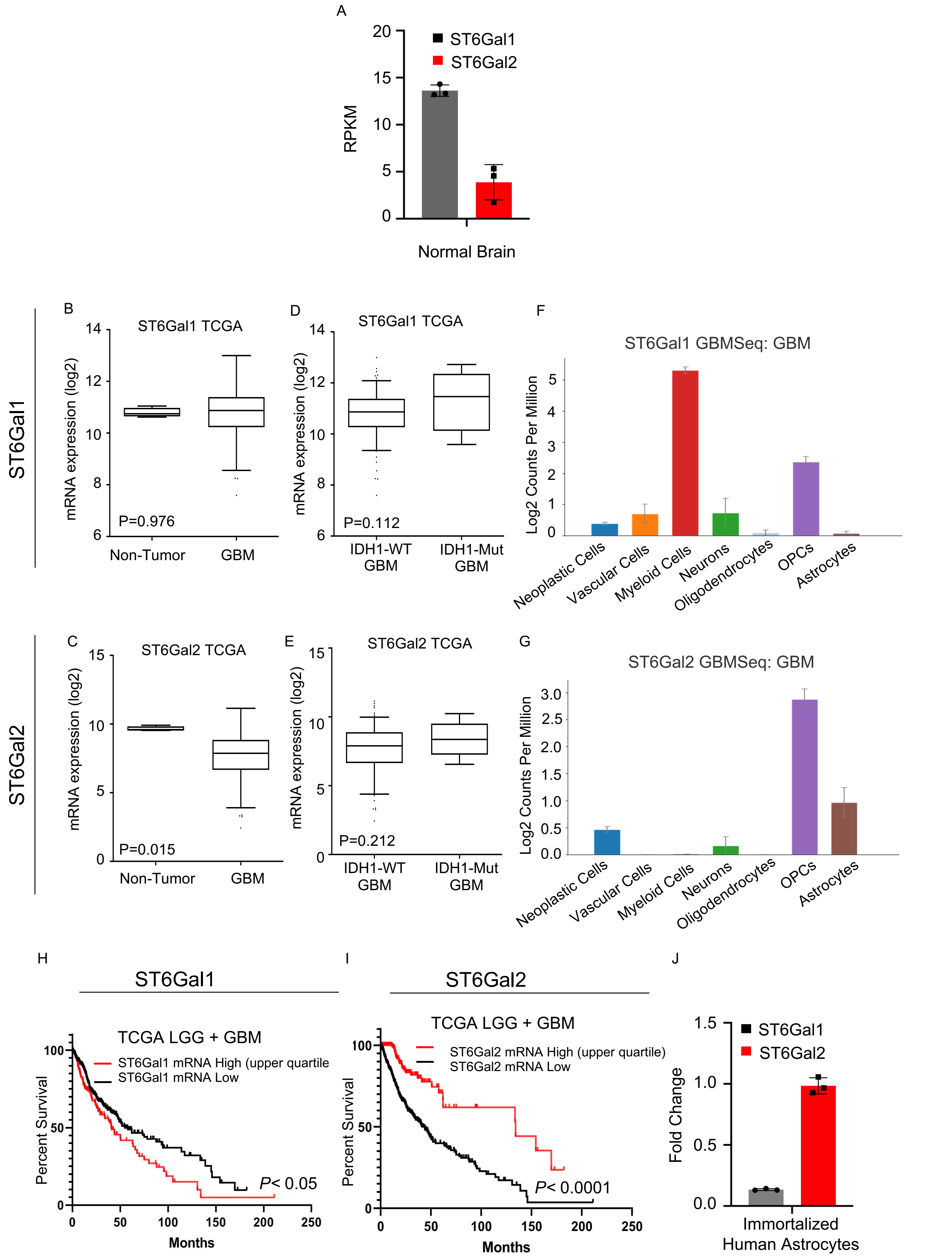
A
ST6Gal1
ST6Gal2
RPKM
20
15
10
5
0
Normal Brain
ST6Gal1
ST6Gal2
B
ST6Gal1 TCGA
mRNA expression (log2)
14
12
10
8
6
P=0.976
Non-Tumor
GBM
C
ST6Gal2 TCGA
mRNA expression (log2)
15
10
5
0
P=0.015
Non-Tumor
GBM
D
ST6Gal1 TCGA
mRNA expression (log2)
P=0.112
IDH1-WT GBM
IDH1-Mut GBM
E
ST6Gal2 TCGA
mRNA expression (log2)
P=0.212
IDH1-WT GBM
IDH1-Mut GBM
F
ST6Gal1 GBMSeq: GBM
Log2 Counts Per Million
Neoplastic Cells
Vascular Cells
Myeloid Cells
Neurons
Oligodendrocytes
OPCs
Astrocytes
G
ST6Gal2 GBMSeq: GBM
Log2 Counts Per Million
3.0
2.5
2.0
1.5
1.0
0.5
0.0
Neoplastic Cells
Vascular Cells
Myeloid Cells
Neurons
Oligodendrocytes
OPCs
Astrocytes
H
ST6Gal1
TCGA LGG + GBM
ST6Gal1 mRNA High (upper quartile
ST6Gal1 mRNA Low
Percent Survival
100
50
0
P< 0.05
0
50
100
150
200
250
Months
I
ST6Gal2
TCGA LGG + GBM
ST6Gal2 mRNA High (upper quartile)
ST6Gal2 mRNA Low
Percent Survival
P< 0.0001
Months
J
ST6Gal1
ST6Gal2
Fold Change
1.5
1.0
0.5
0.0
Immortalized Human Astrocytes

Supplemental Figure 2. Higher ST6Gal1 or lower ST6Gal2 correlates with worse prognosis in all glioma patients. **(A)** Reads Per Kilobase of transcript, per Million mapped reads (RPKM) of ST6Gal1 and ST6Gal2 genes in normal brain tissue as reported in the Human Protein Atlas RNA-sequencing normal tissues project (PRJEB4337). Expression of **(B, D, F)** ST6Gal1 or **(C, E, G)** ST6Gal2 mRNA in the TCGA GBM RNAseq dataset accessed via Gliovis at http://gliovis.bioinfo.cnio.es **(B, D, C, E)** or GBMseq dataset accessed via http://gbmseq.org **(F, G)** was analyzed for **(B, C)** non-tumor tissue in comparison to GBM, **(D, E)** IDHwt GBM vs. IDHmut GBM (p values with Unpaired t-tests are shown), or single cell expression **(F, G)**. Kaplan–Meier survival curves depicting survival of all glioma patients (LGG and GBM) with differential **(H)** ST6Gal1 or **(I)** ST6Gal2 mRNA levels with the upper quartile cutoff. P values shown are for Log-rank and Wilcoxon test. **(J)** RNA levels of ST6Gal1 or ST6Gal2 in immortalized human astrocytes (iHA) grown in media containing FBS demonstrating the ability of the ST6Gal2 primer to detect signal. Fold change is relative to ST6Gal2.

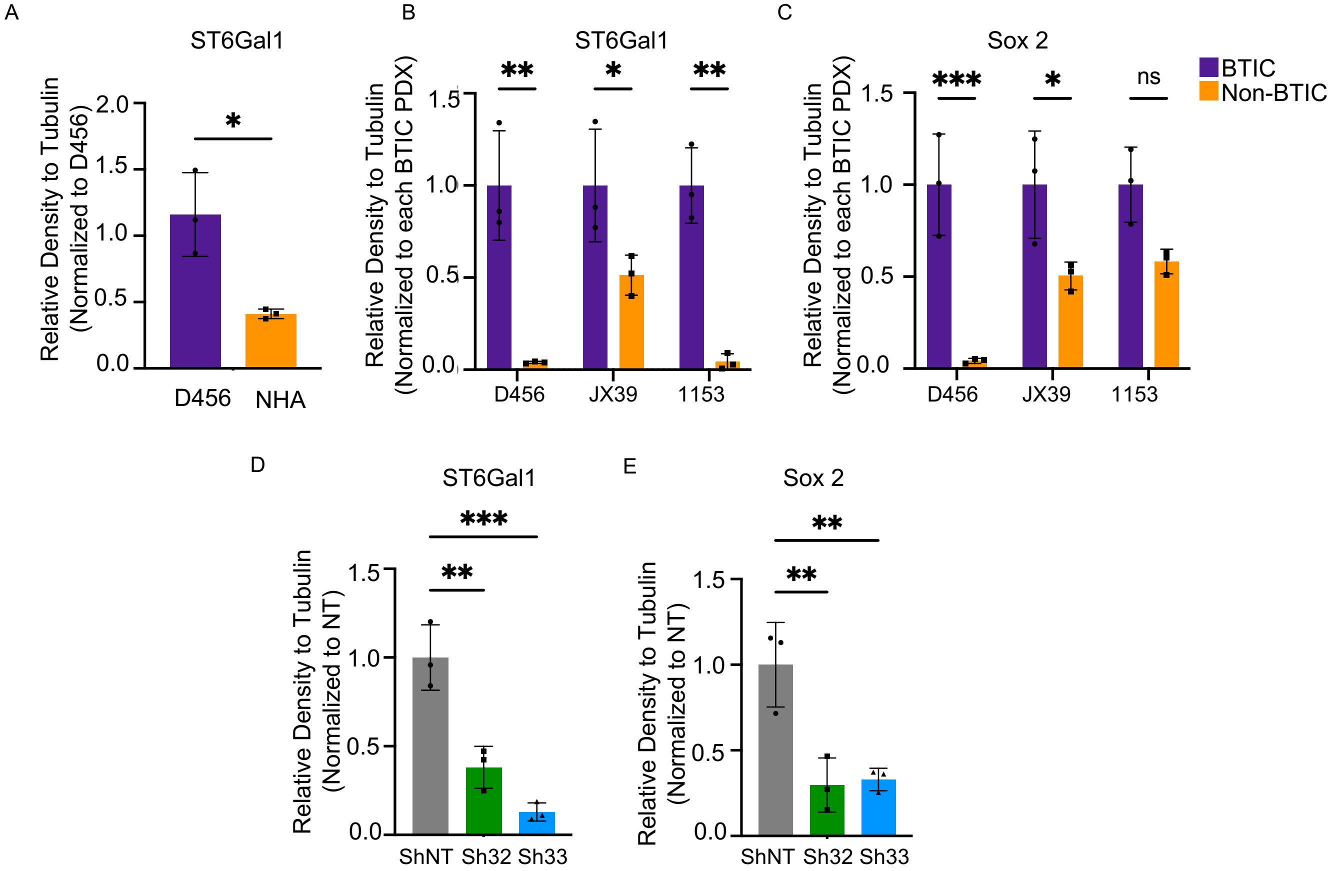

Supplemental Figure 3. Quantification of immunoblots demonstrating elevated ST6Gal1 expression in BTICs. (A) Densitometry values of ST6Gal1 as determined using ImageJ in GBM PDX D456 and non-malignant NHA represented in Figure 2D. Data are displayed as means ± SD. *p<0.05, with independent t-test including biological replicates (N=3). Densitometry values of **(B)** ST6Gal1 and **(C)** Sox 2 as determined using ImageJ in GBM PDXs D456, Jx39 and 1153 grown in medium for BTICs or BTICs differentiated in FBS for 96 hrs represented in figure 2E. Densitometry values of **(D)** ST6Gal1 and **(E)** Sox2 as determined using ImageJ in GBM PDX D456 modulated with lentiviral transduced nontargeting control shRNA (shNT) or two different shRNA constructs targeting ST6Gal1 (sh32 and sh33) represented in figure 2G. **(B-E)** Data are displayed as means ± SD. *P < 0.05; **P < 0.01; 2-way ANOVA with Tukey's multiple comparisons test.

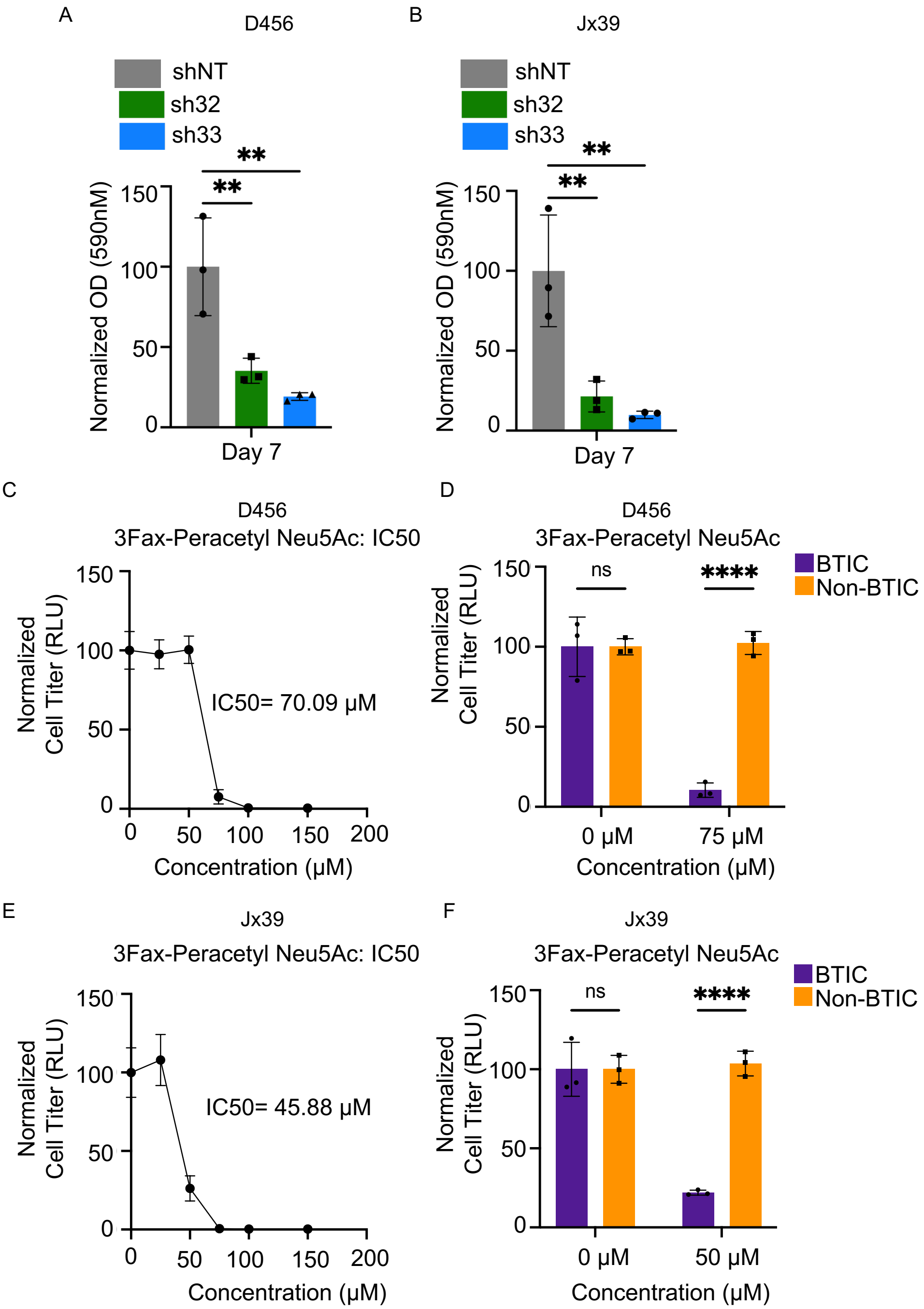

Supplemental Figure 4. Targeting ST6Gal1 and sialyltransferase activity decreases GBM growth. Growth of **(A)** D456 and **(B)** Jx39 BTICs with (sh32, sh33) and without (non-targeting control, shNT) ST6Gal1 was measured at day 14 using crystal violet staining, absorbance at 590 nm. Individual data points are shown with the errors bars as mean ± SD, (n=3). **P* < 0.05; ***P* < 0.01; ****P* < 0.001, 2-way ANOVA with Tukey's multiple comparisons test. The experiments were repeated in three independent biological replicates. Data from one representative experiment are shown. BTICs derived from **(C, D)** D456 or **(E, F)** Jx39 PDX were treated with the indicated concentrations of the non-specific sialyl transferase inhibitor 3Fax-Peracetyl Neu5Ac. IC50 of 3Fax-Peracetyl Neu5Ac for **(C)** D456 and **(E)** Jx39 BTICs was determined using non-linear least squares fit model. **(D)** D456 and **(F)** Jx39 BTICs or non-BTICs (BTICs differentiated via the removal of EGF and FGF and the addition of FBS for 96 hrs) were treated with 0 and 75 µM of 3Fax-Peracetyl Neu5Ac for D456 and **f** 0 and 50 µM of 3Fax-Peracetyl Neu5Ac for Jx39, using DMSO as control. Individual data points are shown with the errors bars as mean ± SD, (n=3). **P* < 0.05; ***P* < 0.01; ****P* < 0.001, 2-way ANOVA with Tukey's multiple comparisons test. The experiments were repeated in three independent biological replicates. (**C-F**) Cells were grown in respective medium with vehicle control or drug for 7 days followed by growth measurement using Cell Titer Glo 2.0.

GC et al., Supplemental Figure 5

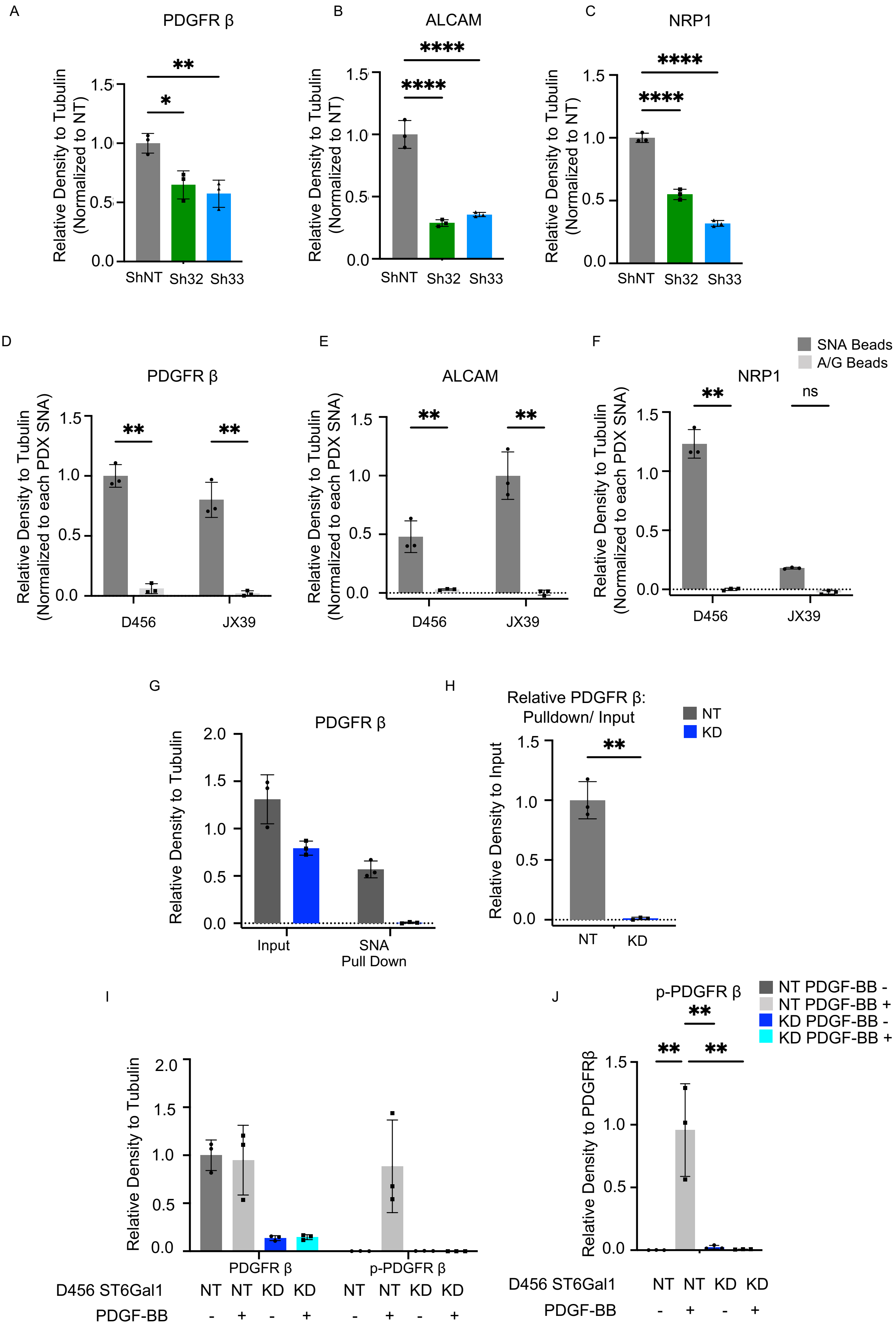

Supplemental Figure 5. ST6Gal1 targeting decreases levels of a subset of N-glycoproteins that are known BTIC regulators. Densitometry values as determined using ImageJ in immunoblotted samples independent of the proteomic analysis for **(A)** PDGFRβ, **(B)** ALCAM, and **(C)** NRP1 protein in figures 4 B, C and D respectively. Densitometry values as determined using ImageJ in GBM PDX D456 for SNA pull down compared to protein A/G bound agarose beads as a control for **(D)** PDGFRβ, **(E)** ALCAM, and **(F)** NRP1 as represented in Figure 4F. **(G)** Densitometry values as determined using ImageJ for SNA pull down of D456 PDX cells with ST6Gal1 KD compared to NT, demonstrating differential pull down of PDGFRβ and **(H)** relative PDGFRβ in SNA pull down fraction compared to input. **(I)** PDGF-BB induced (10 minutes) activation of PDGFRβ in D456 GBM PDX cells with ST6Gal1 KD compared to NT; immunoblotted for p-PDGFRβ and total PDGFRβ. **(J)** Relative p-PDGFRβ normalized with total PDGFRβ expression. Individual data points are shown with the errors bars as mean ± SD, (N=3). *$P < 0.05$; **$P < 0.01$; ***$P < 0.001$, ****$P < 0.0001$, 2-way ANOVA with Tukey's multiple comparisons test.
